# Evaluating different routes of extracellular vesicle administration for cranial therapies

**DOI:** 10.20517/2394-4722.2020.22

**Published:** 2020-06-18

**Authors:** Pericles Ioannides, Erich Giedzinski, Charles L. Limoli

**Affiliations:** Department of Radiation Oncology, University of California, Irvine, CA 92697-2695, USA

**Keywords:** Extracellular vesicles, cranial therapy, cognitive dysfunction

## Abstract

**Aim:**

Human stem cell-derived extracellular vesicles (EV) provide many advantages over cell-based therapies for the treatment of functionally compromised tissue beds and organ sites. Here we aimed to highlight multiple administration routes for the potential treatment of various forms of brain injury.

**Methods:**

Human neural stem cell-derived EV were isolated from conditioned media and administered via three distinct routes: intrahippocampal transplantation, retro-orbital vein injection, and intranasal. EV were administered after which brains were evaluated to determine the capability of EV to translocate into normal tissue.

**Results:**

Data showed no significant differences in the amount of EV able to translocate across the brain, indicating the functional equivalence of each administration route to effectively deliver EV to the brain parenchyma.

**Conclusion:**

Findings show that both systemic administration routes (retro-orbital vein or intranasal delivery) afforded effective penetrance and perfusion of EV throughout the brain in a minimally invasive manner, and point to a translationally tractable option for treating certain neurological disorders including those resulting from cranial irradiation procedures.

## INTRODUCTION

Extracellular vesicles (EV) are secreted by nearly every mammalian cell type and contain a wealth of bioactive cargo capable of modulating target cell function through a variety of paracrine signaling mechanisms^[1]^. Depending on such factors as cellular origin, cargo contents, membrane composition, and target cell indications, interactions of EV with damaged, diseased, or otherwise compromised tissue beds can promote functional recovery^[1,2]^. As membrane-bound vesicles, EV are typically divided into two groups based on size and mode of formation. Microvesicles (MV) tend to be larger (100 nm to 1 μm), and are directly assembled from cellular contents and released by outward budding of the cell membrane^[3]^. Exosomes are smaller (30-100 nm) intraluminal vesicles within endosome-derived multivesicular bodies that then fuse with and release from the plasma membrane^[4]^. For the resolution of radiation injury, no clear evidence has demonstrated a therapeutic advantage of human stem cell-derived MV over exosomes or vice versa, so the EV-based treatments used in this study include the full-size range of vesicles secreted into the conditioned medium by the proliferating human neural stem cells.

Compared to stem cell therapies, the ability of EV to stimulate regenerative healing while eliminating risks of teratoma/tumor formation and confounding complications associated with immune suppression, indicate their potential translational utility. While regenerative approaches for implementing stem cell treatments in the context of radiation injury hold tremendous potential^[5]^, EV circumvents certain stem cell-based caveats due to their low immunogenicity, long-circulating half-life and ability to cross the blood-brain barrier^[2,6,7]^. Recent work from our laboratory has demonstrated the functional equivalence of EV and human stem cells following intra-cranial delivery to the irradiated hippocampus^[8,9]^. These studies demonstrated that both therapies mitigated radiation-induced cognitive dysfunction while preserving host neuronal morphology and attenuating neuroinflammation^[8,9]^. EV-based therapies have also been used to reduce indications of neuroinflammation or cognitive dysfunction associated with chemobrain^[10]^, other neurodegenerative conditions^[11,12]^, and physical injury^[13–15]^, indicating their widespread tolerance and broad efficacy in the brain.

Migration of EV through the extracellular space or circulation provides the routes whereby EV can interact with target cells, presumably through interactions between transmembrane proteins on the EV and specific receptors on the surface of the target cell. Recipient cells internalize EV via either fusion with the plasma membrane or more commonly by endocytosis^[16]^. This then initiates the functional transfer of critical bioactive cargo containing lipids, proteins, organelles, and an assortment of nucleic acids including microRNA (miRNA). The ability of EV to target and functionally interact within the radiation-injured tissue bed provides a heretofore unexplored area for resolving a wide range of dose-limiting normal tissue toxicities associated with the radiotherapeutic management of cancer. For these reasons we embarked on a targeted technical study to evaluate whether other non-surgical administration routes could deliver hNSC-derived EV to the parenchyma of the brain.

## METHODS

### Stem cell culture, isolation, and labeling of EV

Growth, culturing and maintenance of human neural stem cells (hNSC, H9-derived ENstem-A, Millipore) was approved by the Institutional Human Stem Cell Research Oversight (HSCRO, #2007-5629) and Institutional Biosafety Committees. The growth of hNSC and harvest of conditioned medium from hNSC has been described previously^[9,10]^. All cultures were tested and found to be negative for mycoplasma with MycoAlert Mycoplasma Detection Kit (Lonza, Cat# LT07-118; Basel, Switzerland).

For *in vivo* tracking, EV were labeled with PKH26 (Sigma-Aldrich, Cat# PKH26PCL; St. Louis, MO) the day before transplantation. The EV were then resuspended in Diluent C and incubated with Dye Solution for 2 min with intermittent mixing as per the manufacturers protocol. The dye was quenched with 1% bovine serum albumin in water, and EV were isolated through ultracentrifugation and washed as described^[17]^.

### Administration of EV

All animal experimentation described in this study was per the guidelines provided by the NIH and approved by all Institutional Animal Care and Use Committees (IACUC #AUP-18-032). Male wild type mice (C57BI6/J, Jackson) were maintained in standard housing conditions, respectively (20 °C ± 1 °C; 70% ± 10% humidity; 12 h:12 h light and dark cycle) and had free access to standard rodent chow and water. Four-month-old wild-type C57Bl/6J male mice were divided into the following three groups receiving EV: Intracranial (IC), retro-orbital (RO), and intranasal (IN). For each route of administration, a total of 6.70 × 10^6^ EV were delivered in a total volume of 8 μL (4 sites), 50 μL and 20 μL for IC, RO and IN respectively.

#### IC delivery:

Mice were anesthetized (4%) and maintained on 2% (v/v) isoflurane/oxygen for the stereotaxic implantation of EV. Surgical injections were performed using a 32G microsyringe at an injection rate of 0.25 μL/min. Each hippocampus received 2 distinct injections of EV per hemisphere in an injection volume of 2 μL (EV in sterile hibernation buffer) per site for a total of 4 injections (8 μL) per animal. Stereotactic coordinates from the bregma were anterior-posterior (AP): −1.94, mediolateral (ML): −1.25, dorsal-ventral (DV): −1.50 for the first site and AP: −2.60, ML: −2.0, and DV: −1.5 for the second site.

#### RO delivery:

Mice were anesthetized with 4% isoflurane and gentle pressure was applied using the fingers against the skin that is ventral and dorsal to the eye. A dermal syringe with a 29G needle, bevel down, was inserted gently to the retro-orbital vein by the corner of the eye. The 50 μL EV injection was applied in a slow, smooth fashion. The needle was carefully removed and the mouse was monitored until fully recovered (within 3-5 min) from anesthesia.

#### IN delivery:

Mice were lightly anesthetized (2% isoflurane) and prepared for IN administration of EV^[18]^. Manual restraint was used to hold the mouse in a supine position with the head elevated. The end of a 20 μL micropipette was placed at the external nares, and then the 20 μL solution was poured in slowly by the nasal tip. Mice were held for about 10 s before returning to the holding cage to recover.

### Intracranial imaging

Two days post-EV treatment, mice were deeply anesthetized using isoflurane and euthanized via intracardiac perfusion using 4% paraformaldehyde (Sigma) in 100 mmol/L phosphate-buffered saline (PBS; pH 7.4, Gibco). Brains were cryoprotected using a sucrose gradient (10%-30%) and sectioned coronally into 30 μm thick sections using a cryostat (Microm, Thermo Scientific, US). For each endpoint 4 representative coronal brain sections from each experimental group were selected at approximately 15 section intervals to encompass the rostrocaudal axis from the middle of hippocampus including regions of the prefrontal cortex (PFC), the subventricular zone (SVZ) and the dentate gyrus (DG), which were then stored in PBS. Tissues (*n* = 54) were DAPI nuclear counterstained mounted onto slides and sealed in slow fade/antifade mounting medium (Life Technologies). Confocal analyses were carried out using multiple Z-stacks taken at 5-mm intervals using a confocal laser scanning microscope (Nikon Eclipse TE2000-U, EZ-C2 interface). Individual Z-sections were then analyzed using Nikon Elements software (version 3.0). Images were deconvoluted using AutoQuant X3 and surface analysis was performed with Imaris (v8.5, BitPlane, Inc., Switzerland).

#### Statistical analysis

Statistical analyses were carried out using GraphPad Prism (v6) software. One-way analyses of variance (ANOVA) were used to assess significance between the control, irradiated, and irradiated group receiving EV. When overall group effects were found to be statistically significant, a Bonferroni’s multiple comparisons test was used to compare the 9 Gy with individual experimental groups. Data in the text are presented as means ± SEM, and all analyses considered a value of *P* < 0.05 to be statistically significant.

## RESULTS

### Extensive migration of EV in the host brain via distinct administration routes

Past data has shown that intracranial grafting of hNSC-derived EV affords significant neurological improvements in the irradiated brain^[9]^. In that work, cranial irradiation was associated with significant behavioral deficits that were ameliorated by EV treatments. The neurological benefits of hNSC-derived EV grafted into the hippocampus prompted efforts to determine whether alternative (and non-surgical) routes of administration would suffice for the delivery of EV to the parenchyma of the brain. EV derived from a single batch were administered to mice via IC, RO, and IN routes, after which distinct brain regions (PFC, SVZ, DG) were imaged 2 days following treatments to assess brain penetrance of EV delivered through each route.

**Figure 1.**
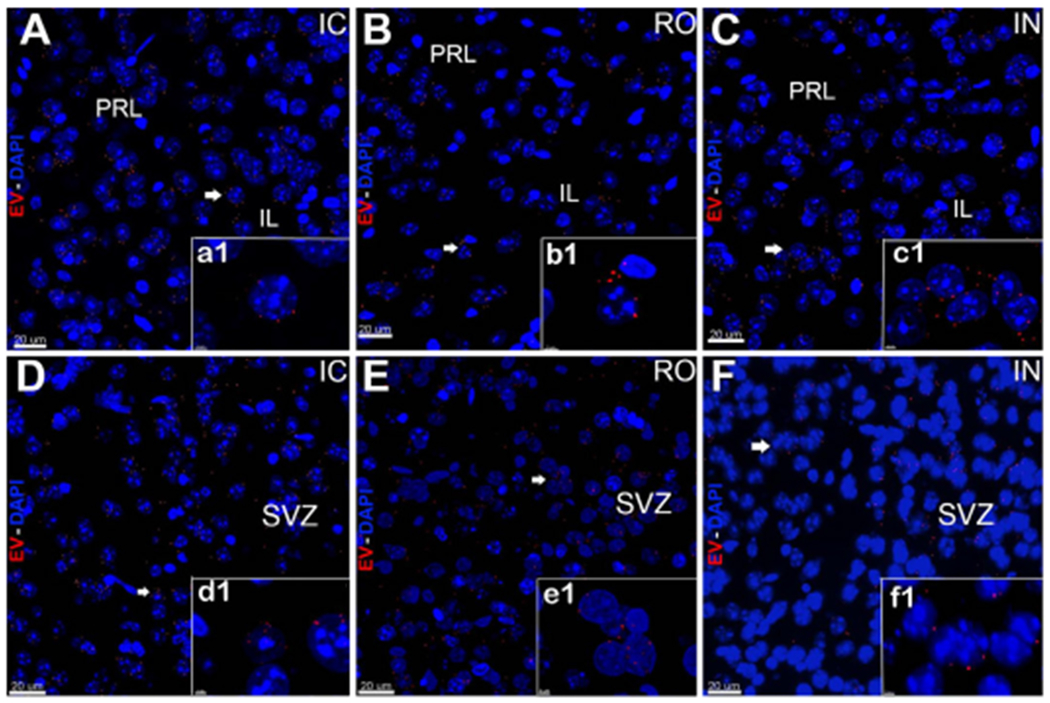
*In vivo* tracking of extracellular vesicles (EV) in the prefrontal cortex and subventricular zone after intracranial (IC), retro-orbital (RO), or intranasal (IN) injections. hNSC-derived-EV labeled with fluorescent dye were transplanted using stereotaxic IC (A, D), RO (B, E), or IN (C, F) injections. The brain tissues were fixed at 48 h post-surgery and sections imaged using confocal microscopy. Confocal Z-stacks were collected at 60 × magnification and qualitatively demonstrate that injected EV (red, DAPI nuclear counterstain, blue) migrated to the pre-limbic (PRL) and infra (IL) limbic structures of the PFC (A-C) and the SVZ (D-F). Magnified images (a1-f1) demonstrate localization of EV in close vicinity of the cell bodies after IC, RO, and IN administration. Scale bars: 20 μm (A-F) and 3 μm (a1-f1)

**Figure 2.**
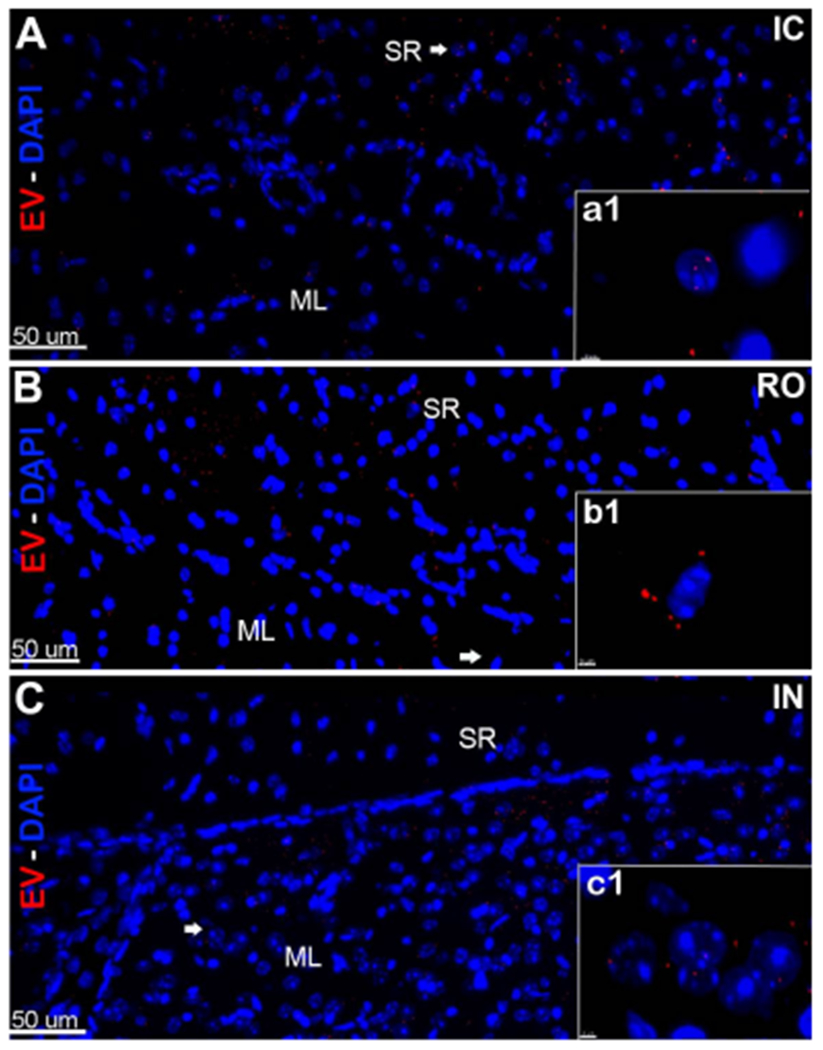
*In vivo* tracking of extracellular vesicles (EV) in the hippocampus after intracranial (IC), retro-orbital (RO), or intranasal (IN) injections. hNSC-derived EV labeled with fluorescent dye were transplanted using stereotaxic IC (A), RO (B), or IN (C) injections. Brain tissues were fixed at 48 h post-treatment, sections were imaged using confocal microscopy and Z-stacks were collected at 60 magnification. Fluorescently-labeled EV (red; DAPI nuclear counter-stain, blue) were located and migrated through the CA1 stratum radiatum (SR) and granule cell molecular layers (ML) in the host hippocampus. Magnification (a1-c1) demonstrates the close vicinity of EV around the cell bodies after IC, RO, and IN administration. Scale bars: 50 μm (A-C) and 3 μm (a1-c1)

**Figure 3.**
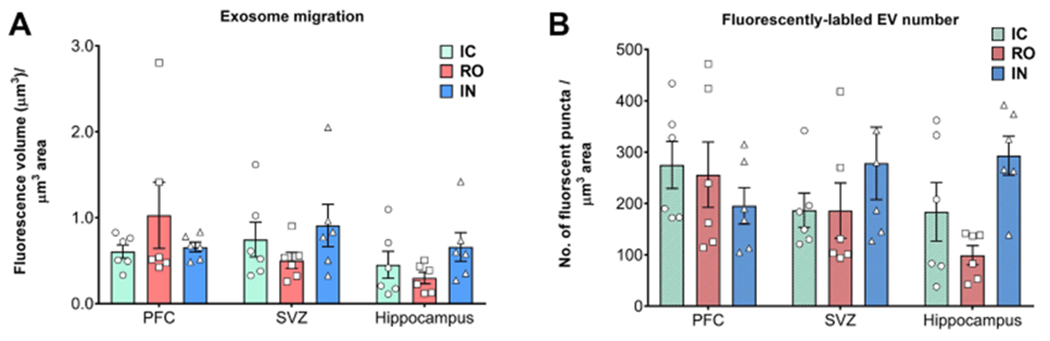
Quantification of extracellular vesicles (EV) throughout the brain. The volume of EV fluorescence intensity (A) or the yield of fluorescent EV puncta plotted (B) as a function of administration route reveal the relatively equal and widespread distribution of EV throughout the prefrontal cortex (PFC), subventricular zone (SVZ), and hippocampus. Differences between the yields of EV quantified between administration route and brain subregion were not found to be statistically different

Compared to IC grafting, data demonstrated that systemic delivery routes provided comparable doses of EV to the brain [Figures 1 and 2]. Intracranial grafting of EV [Figure 1A and D], showed equivalent levels when compared to the brains in which EV were injected RO [Figure 1B and E] or delivered IN [Figure 1C and F] in the PFC [Figure 1A–C] or the SVC [Figure 1D–F]. Similar observations were obtained from comparisons of EV content for the hippocampal DG following each administration route [Figure 2]. Quantification of EV fluorescence between the different administration routes and/or subregions of the brain did not reveal consistent trends indicating that one delivery route was more or less efficacious than another [Figure 3A]. Similar findings were obtained when the number of fluorescent EV puncta were quantified throughout the same brain regions [Figure 3B]. Delivery of EV via IC, RO, and IN routes were all found to penetrate the different subregions of the brain at roughly equivalent levels, where differences found between either method of quantification did not reach statistical significance [Figure 3]. These findings corroborate our past data, where comparable distributions of EV were found between ipsi- and contra-lateral sites when delivered via unilateral IC route^[19]^. Current data indicate that qualitatively similar yields and widespread distribution of EV can be obtained throughout the brain using various administration routes.

## DISCUSSION

While certain applications of EV-based therapies have begun, their potential for the resolution of radiation-induced normal tissue toxicities remains relatively unexplored. Our past work demonstrating the neuroprotective benefits of cranially grafted EV, when substituted for stem cells, into the irradiated brain laid the foundation for much of the current work. The ability of EV to ameliorate radiation-induced cognitive dysfunction is noteworthy if not remarkable, especially given that a single treatment via cranial graft was successful in reducing serious and multifaceted normal tissue complications associated with the radiotherapeutic management of brain cancer. Importantly, we have now demonstrated the feasibility of delivering EV through non-surgical routes, thereby providing a more tractable and appealing alternative for translating EV therapies to the clinic.

Our current study was designed to advance potential therapeutic applications of EV, by demonstrating the practical feasibility of delivering EV through multiple routes. While EV surface markers and the content will greatly dictate *in vivo* targeting and efficacy, specifics related to disease, insult, and/or injury will largely dictate whether a targeted or systemic administration will provide the most optimal treatment strategy. Furthermore, to avoid any possible confounding impact on concurrent cancer treatments, we envision that such treatments would transpire after the cessation of radiotherapy and/or chemotherapy. This proposition is also supported by recent evidence demonstrating that irradiation can significantly alter the protein, lipid, and miRNA cargo of EV derived from cancer and normal cells and circulating EV found within the plasma^[20–22]^.

In any case, this targeted study demonstrates the potential feasibility of administering EV through systemic routes, thereby avoiding the requirement for more invasive surgical procedures. The equivalence found between administration routes for delivering EV to various subregions of the brain is provocative but not without caveats. Inherent uncertainties are associated with comparisons of these data, as the different EV administration routes selected lead to variable (and unavoidable) sample dilution *in vivo.* Importantly, while the net amount of EV between each treatment was held constant, each administration route necessitated different volumes for proper biological distribution. For instance, IC injections cannot accommodate volumes over 2 μL/site and systemic injections (RO at 50 μL, IN at 25 μL) require larger boluses to facilitate more homogeneous delivery. Notwithstanding, further work is still required to more rigorously validate whether systemic administration of EV affords functionally equivalent neuroprotection to the otherwise compromised or irradiated CNS.

So where does the field of EV therapy stand for the treatment of radiation and other normal tissue toxicities? Future studies should seek to define optimal cellular sources of EV to delineate the mechanism of action, to identify bioactive cargo, and to pinpoint efficacious EV dosing regimens. While current data points to several possible options for delivering EV to the brain, in humans, intravenous routes are likely to provide the best combination of widespread availability and feasibility for repeated treatment regimens. Clearly, a more systematic and complete characterization of EV surface markers and the content will be required to translate these approaches to the clinic and be necessary to evaluate other potential risks. While the lack of teratoma formation and reduced immunogenic response inherent to EV therapies are clear benefits, certain safety issues remain to be thoroughly addressed, especially in the area of cancer treatments. Further work must determine whether such approaches activate “cold” or latent cancers or alter the growth of recurrent malignancies when administered after the cessation of specific cancer treatments. Despite the caveats associated with any burgeoning therapy, EV provide a potentially attractive therapeutic avenue for resolving normal tissue toxicities associated with radiotherapy, injury, disease, and aging. Studies here provide the proof of principle highlighting the tremendous potential of EV-based therapy and underscore that such pursuits are warranted.
